# Heat and Acupuncture to Manage Osteoarthritis of the Knee (HARMOKnee): Protocol for an Effectiveness-Implementation Hybrid Randomized Controlled Trial

**DOI:** 10.2196/54352

**Published:** 2024-04-03

**Authors:** Bryan Yijia Tan, Chun Yue Tan, Tong Leng Tan, Su-Yin Yang, Gillian Long Szu Chew, Siang Ing Tan, Yu Chun Chua, Yew Wai Yan, Derrick Bing Quan Soh, Tong Hwee Goh, Pu Jue Ng, Yee Teck Ng, Swee Boey Kuan, Bo Siang Teo, Keng He Kong, Michelle Jessica Pereira, Hui Ping Ng

**Affiliations:** 1 Department of Orthopaedic Surgery Woodlands Health National Healthcare Group Singapore Singapore; 2 Department of Orthopaedic Surgery Tan Tock Seng Hospital National Healthcare Group Singapore Singapore; 3 Psychology Service Woodlands Health Singapore Singapore; 4 Complementary Integrative Medicine Tan Tock Seng Hospital National Healthcare Group Singapore Singapore; 5 Traditional Chinese Medicine Pain Management, Medical Department Singapore Chung Hwa Medical Institution Singapore Singapore; 6 Teaching Department Singapore College of Traditional Chinese Medicine Singapore Singapore; 7 Rehabilitation Centre Tan Tock Seng Hospital National Healthcare Group Singapore Singapore; 8 Health Services and Outcomes Research National Healthcare Group Singapore Singapore

**Keywords:** knee osteoarthritis, acupuncture, heat therapy, effectiveness-implementation hybrid study, randomized controlled trial, RCT

## Abstract

**Background:**

Knee osteoarthritis (KOA) is one of most prevalent and fastest-growing causes of pain, impaired mobility, and poor quality of life in the rapidly aging population worldwide. There is a lack of high-quality evidence on the efficacy of traditional Chinese medicine (TCM), particularly acupuncture, and a lack of KOA practice guidelines that are tailored to unique population demographics and tropical climates.

**Objective:**

Our HARMOKnee (Heat and Acupuncture to Manage Osteoarthritis of the Knee) trial aims to address these gaps by evaluating the short- and medium-term clinical and cost-effectiveness of acupuncture with heat therapy in addition to standard care, compared to standard care alone. Through a robust process and economic evaluation, we aim to inform evidence-based practice for patients with KOA to facilitate the large-scale implementation of a comprehensive and holistic model of care that harmonizes elements of Western medicine and TCM. We hypothesize that acupuncture with heat therapy as an adjunct to standard care is clinically more effective than standard care alone.

**Methods:**

A multicenter, pragmatic, parallel-arm, single-blinded, effectiveness-implementation hybrid randomized controlled trial will be conducted. We intend to recruit 100 patients with KOA randomized to either the control arm (standard care only) or intervention arm (acupuncture with heat therapy, in addition to standard care). The inclusion criteria are being a community ambulator and having primary KOA, excluding patients with secondary arthritis or previous knee replacements. The primary outcome measure is the Knee Osteoarthritis Outcome Score at 6 weeks. Secondary outcome measures include psychological, physical, quality of life, satisfaction, and global outcome measures at 6, 12, and 26 weeks. A mixed method approach through an embedded process evaluation will facilitate large-scale implementation. An economic evaluation will be performed to assess financial sustainability.

**Results:**

Patient enrollment has been ongoing since August 2022. The recruitment process is anticipated to conclude by July 2024, and the findings will be analyzed and publicized as they are obtained. As of November 6, 2023, our patient enrollment stands at 65 individuals.

**Conclusions:**

The findings of our HARMOKnee study will contribute substantial evidence to the current body of literature regarding the effectiveness of acupuncture treatment for KOA. Additionally, we aim to facilitate the creation of standardized national guidelines for evidence-based practice that are specifically tailored to our unique population demographics. Furthermore, we seek to promote the adoption and integration of acupuncture and heat therapy into existing treatment models.

**Trial Registration:**

ClinicalTrials.gov NCT05507619; https://clinicaltrials.gov/study/NCT05507619

**International Registered Report Identifier (IRRID):**

DERR1-10.2196/54352

## Introduction

Knee osteoarthritis (KOA) is an age-related degeneration representing “wear and tear” of the knee joint, and it is one of the most prevalent and fast-growing causes of pain, impaired mobility, and poor quality of life (QOL) in the rapidly aging population worldwide [1]. The 2019 Global Burden of Disease Study reported that the knee is the joint most frequently affected by osteoarthritis, with a prevalence of approximately 365 million, followed by the hand and the hip [2].

Acupuncture is the most prevalent form of traditional medicine practice, with 183 of 202 (90.5%) surveyed countries recognizing its use, as reported by the World Federation of Acupuncture-Moxibustion Societies [3]. A bibliometric analysis reported that of 2189 positive recommendations for the use of acupuncture, about 68% were associated with 107 pain indications [4].

A review of 10 randomized controlled trials (RCTs) concluded that acupuncture is an effective treatment for KOA-related pain and physical dysfunction [5]. However, major updated clinical guidelines from the American Academy of Orthopaedic Surgeons, Joint Surgery Branch of the Chinese Orthopaedic Association, American College of Rheumatology/Arthritis Foundation, and others still consider acupuncture treatment to be insufficient, limited, conditional, or unrecommended for KOA [6]. A review highlighted the lack of evidence supporting the efficacy and cost-effectiveness of acupuncture in osteoarthritis due to poor methodological quality, significant heterogeneity, inadequate reporting of acupuncture treatment details, small sample sizes, and high placebo responses in studies [7].

The primary aim of our study is to evaluate the short- and medium-term clinical effectiveness of acupuncture, part of traditional Chinese medicine (TCM), with far-infrared heat therapy in addition to standard care, compared to standard care alone. The secondary aims include (1) performing a process evaluation to understand the context and identify mechanisms of impact that will inform large-scale implementation of the intervention and (2) performing an economic evaluation to assess the cost-effectiveness of the intervention. We hypothesize that acupuncture with heat therapy as an adjunct to standard care is clinically more effective than standard care alone.

## Methods

### Design

The study is an effectiveness-implementation hybrid trial, which combines effectiveness and implementation components [8,9]. This will be a type 1 hybrid trial where the emphasis and primary aim is to evaluate the effectiveness of acupuncture with far-infrared heat therapy as an adjunct to standard care through a pragmatic RCT under real-world conditions, and its secondary aim is to understand the context of implementation through a mixed-method, process-oriented approach. The pragmatic nature of the study was guided by the Pragmatic Explanatory Continuum Indicator Summary (PRECIS-2) tool [10].

The study will be conducted as a multicenter, pragmatic, parallel-arm, single-blinded RCT using a mixed method approach comparing the clinical effectiveness of acupuncture with far-infrared heat therapy as an adjunct to standard care and standard care alone. The Standard Protocol Items: Recommendations for Interventional Trials (SPIRIT) [11] and the Osteoarthritis Research Society International (OARSI) clinical trial recommendation on the design and conduct of clinical trials for KOA [12] guided the development of the trial protocol. To ensure clear reporting of acupuncture treatment details, Standards for Reporting Interventions in Clinical Trials of Acupuncture (STRICTA) reporting guidelines [13] will also be followed. The findings of the trial will be reported according to the Consolidated Standards of Reporting Trials (CONSORT) 2010 [14] guidelines for reporting parallel-group randomized trials. The study will use an explanatory sequential mixed method design where the qualitative data from interviews will be used to interpret and provide context for the quantitative results.

### Participants

We will recruit patients based on the eligibility criteria outlined in Textbox 1.

**Eligibility criteria.**

**Inclusion criteria**

- National Institute of Health and Care Excellence (NICE) clinical criteria for knee osteoarthritis (KOA) [5]:
  Being aged 45 years or older
  Having activity-related joint pain
  Having either no morning joint-related stiffness or morning stiffness that lasts no longer than 30 minutes
- Being a community ambulator with or without walking aid

**Exclusion criteria**

- Alternative diagnosis to KOA, such as referred pain from the spine or hip
- Other forms of arthritis, such as inflammatory arthritis
- Inability to comply with study protocol (eg, due to dementia)
- Previous knee arthroplasty
- Being a wheelchair user
- Medical conditions that would medically interfere with study involvement, such as decompensated heart failure, stroke, and end-stage renal failure
- Allergies to metal (needles)

### Components of the Study

The Heat and Acupuncture to Manage Osteoarthritis of the Knee (HARMOKnee) study will have three components: (1) an RCT, (2) an economic evaluation, and (3) a process evaluation.

### Component 1: RCT

This RCT will be administered in accordance with the STRICTA guidelines, an extension of the CONSORT statement designed to enhance the quality of clinical trials of acupuncture [13].

#### Trial Procedure and Recruitment

**Figure 1:**
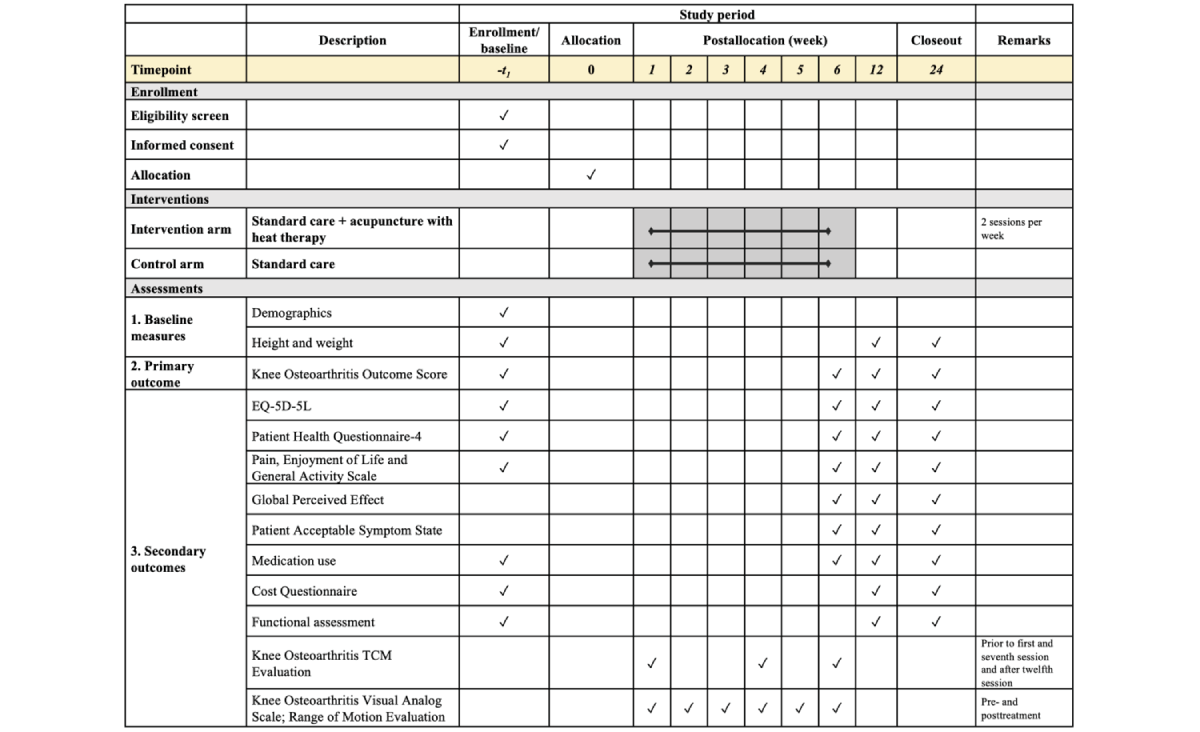
Standard Protocol Items: Recommendations for Interventional Trials (SPIRIT) figure of the study schedule. TCM: traditional Chinese medicine.

**Figure 2:**
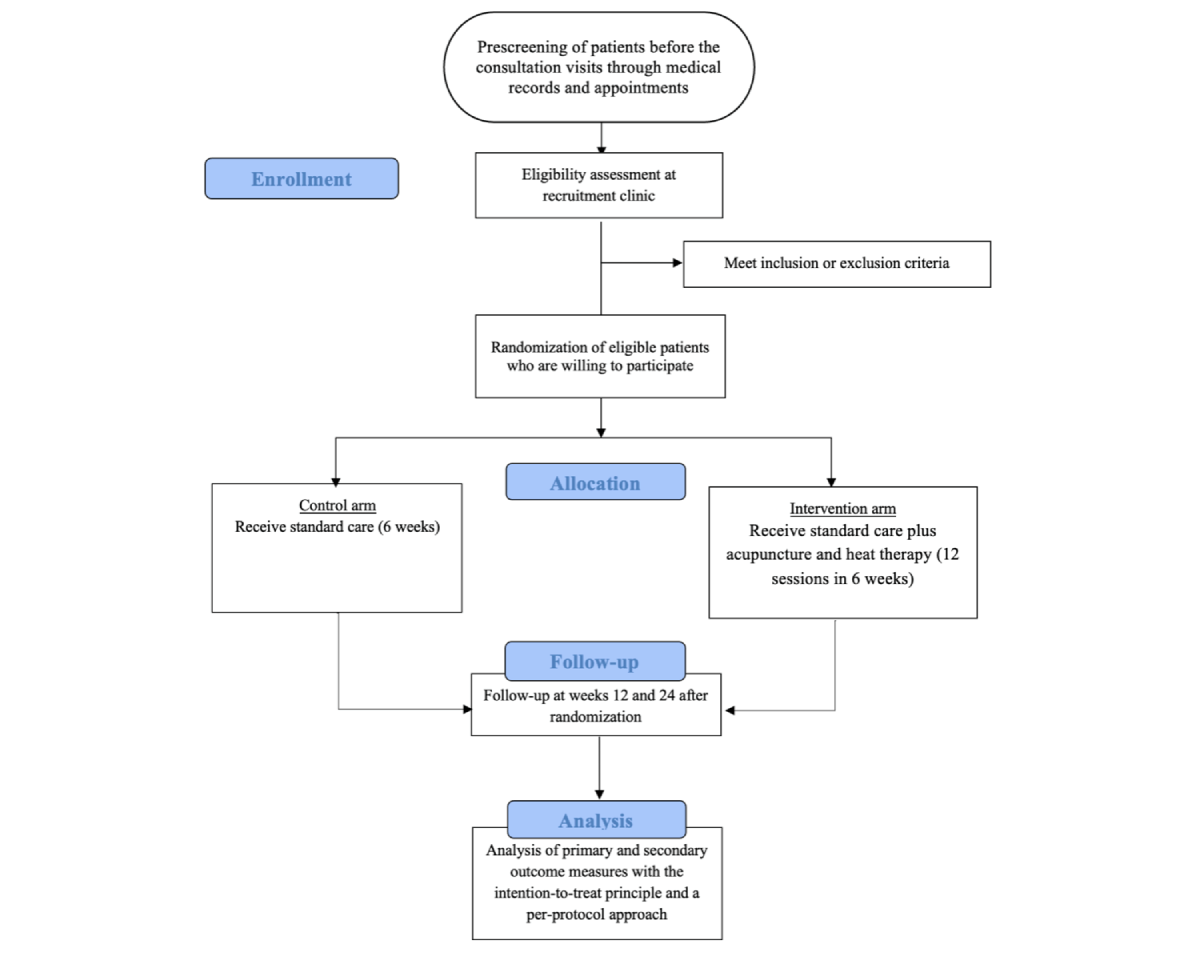
Consolidated Standards of Reporting Trials (CONSORT) flowchart of the study.

By using the hospital’s electronic medical records and appointment system, patients who are referred to the orthopedic surgery outpatient clinic at a tertiary hospital in Singapore will be screened based on the eligibility criteria prior to their actual consultation visit. During the clinic visit, potential patients will be invited to participate in the study if they meet all the inclusion and exclusion criteria. A research assistant will share details of the study with patients who are interested. Written informed consent will be obtained for those who subsequently agree to join the trial. The reasons for rejection to participate will be recorded. Each consenting participant will be asked to complete the baseline measures. Patients will then be randomized into the control arm or the intervention arm. Considering previous studies [15,16], patients who have received TCM treatment within 1 week prior to the trial will be subject to a 2-week washout period, counting from their last TCM treatment date. They will be requested to stop any form of acupuncture before beginning the trial. For this group of patients, randomization and baseline measures will be performed after the wash-out period. Participants are allowed to take any analgesics as needed throughout the study duration, and this will be recorded. All medications (prescription and over the counter), vitamin and mineral supplements, and herbs taken by the participant will be documented. Data collection will be done at baseline and weeks 6, 12, and 26. The research assistant will follow up with the participants regularly to ensure study completion. Figures 1 and 2 illustrate the study schedule of enrollment, interventions, and outcome measurements according to the SPIRIT guidelines and the CONSORT flowchart, respectively.

#### Randomization and Concealment of Allocation

Patients who consent to participate will be randomized in a 1:1 allocation ratio to intervention or standard care using a stratified, permuted, block randomization method (block sizes of 4, 6, and 8). Stratification is based on gender with a female to male ratio of 2:1. The unbalance ratio was based on the World Health Organization’s estimate of the prevalence of symptomatic osteoarthritis for people aged 60 years or older, among whom the prevalence of women is almost double that of men (male vs female: 9.6% vs 18%) [17]. The allocation sequence is generated by an independent statistician a priori using Stata (version 16.1; StataCorp) and will be kept concealed from the study team. Randomization will be done using the REDCap (Research Electronic Data Capture; Vanderbilt University) randomization module based on the allocation sequence; allocation will be locked once assigned. Randomization and intervention allocation will only be performed by the study team after the patient is counselled fully about the study and provides informed consent.

#### Intervention: Acupuncture Plus Heat Therapy and Standard Care

After reviewing international literature and engaging with local TCM experts, this clinical intervention was designed in accordance with local practice. The intervention involves acupuncture with far-infrared heat therapy in addition to standard care, which will be administered by qualified TCM practitioners from Tan Tock Seng Hospital’s Complementary Integrative Medicine Clinic (CIMC) and Singapore Chung Hwa Medical Institution (SCHMI), who have full registration with the Traditional Chinese Medicine Practitioners’ Board and a minimum of 3 years of acupuncture practice. Before the commencement of the trial, all the acupuncturists from the participating institutions will undergo training on acupoint locations and needling methods to ensure standardization of acupuncture techniques in accordance to the acupuncture protocol that was developed by consensus among all the senior acupuncturists participating in this trial. The treatment protocol and rationale are elaborated below.

Our study aims to evaluate the therapeutic effectiveness of acupuncture combined with far-infrared heat therapy in addition to standard care, compared to standard care alone. To achieve this purpose, we have not included sham acupuncture as an inactive control in our study design. There are controversies about the validity of sham acupuncture. A study found that sham acupuncture may produce comparable effects on biomarkers as *verum* acupuncture, which suggests that sham acupuncture, being an inactive intervention akin to a placebo, should be reevaluated [18]. Additionally, heat therapy will yield beneficial outcomes in the treatment, regardless of whether *verum* or sham acupuncture is used.

#### Acupuncture Intervention Rationale

Although the 2007 Singapore Ministry of Health clinical guidelines for KOA recommend electroacupuncture as an adjunct therapy, anecdotal input from our local subject matter experts suggests that some patients are afraid of electrical stimulation and that it is generally less well tolerated due to the pain of electrical stimulation. Studies have also shown that even though electroacupuncture has advantages compared to standard care, acupuncture with heat therapy, a more tolerable option, possesses similar efficacy as electroacupuncture when combined with the usual pharmacotherapies [19]. Therefore, we chose to evaluate acupuncture with heat therapy to establish its clinical effectiveness as an adjunctive therapy in the management of KOA, with long-term implementability a key consideration.

Upon careful review of the evidence-based Guidelines of Clinical Practice with Acupuncture and Moxibustion for KOA [20] and the intervention protocols of several acupuncture RCTs that examined patients with KOA (Multimedia Appendix 1, Figure S1 [10, 21-29]), acupoints that were previously reported to be effective in pain relief and improving knee flexibility were identified.

Guided by international evidence in consultation with the local TCM collaborators, a consensus on a set of proximal and distal acupoints based on TCM meridian differentiation was reached (Multimedia Appendix 1, Figure S2 [30,31]).

#### Acupoint Selection

**Figure 3:**
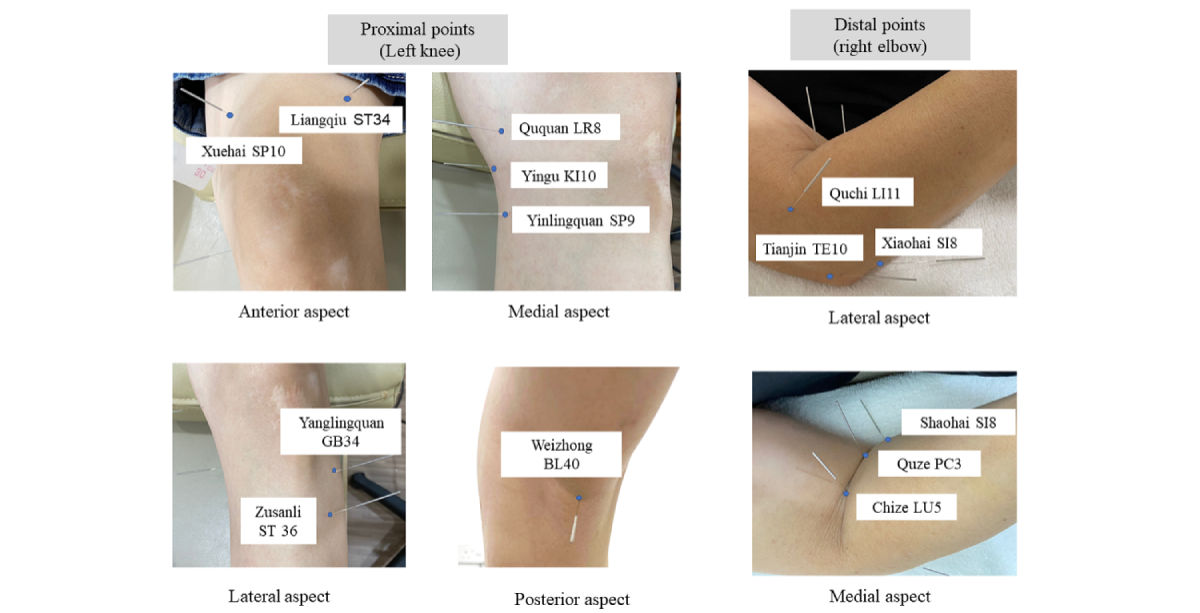
Proximal and distal acupoints.

In total, 14 needle insertions will be made on a fixed set of proximal and distal acupoints for each patient per session (Figure 3).

Distal acupoints will be applied on the opposite elbow [32] to increase the analgesic efficacy according to contralateral collateral needling (缪刺) from *Huang Di Nei Jing* (*Yellow Emperor's Inner Classic* in English). It is a therapy where the right side is acupunctured if the left side is diseased and vice versa; the upper side is acupunctured if the below is diseased and vice versa. Studies have shown that contralateral collateral needling unblocks collaterals and is potentially more effective than conventional acupuncture for some conditions, including hemiplegia following acute ischemic stroke, cervical shoulder pain, insomnia, and KOA [33-35]. From a pragmatic perspective, patients with bilateral knee pain will only receive acupuncture on the most painful knee and the opposite elbow. This practice is supported with evidence that suggests unilateral acupuncture is equally as effective as bilateral acupuncture in increasing function and reducing the pain associated with KOA [36].

#### Treatment Regimen

**Figure 4:**
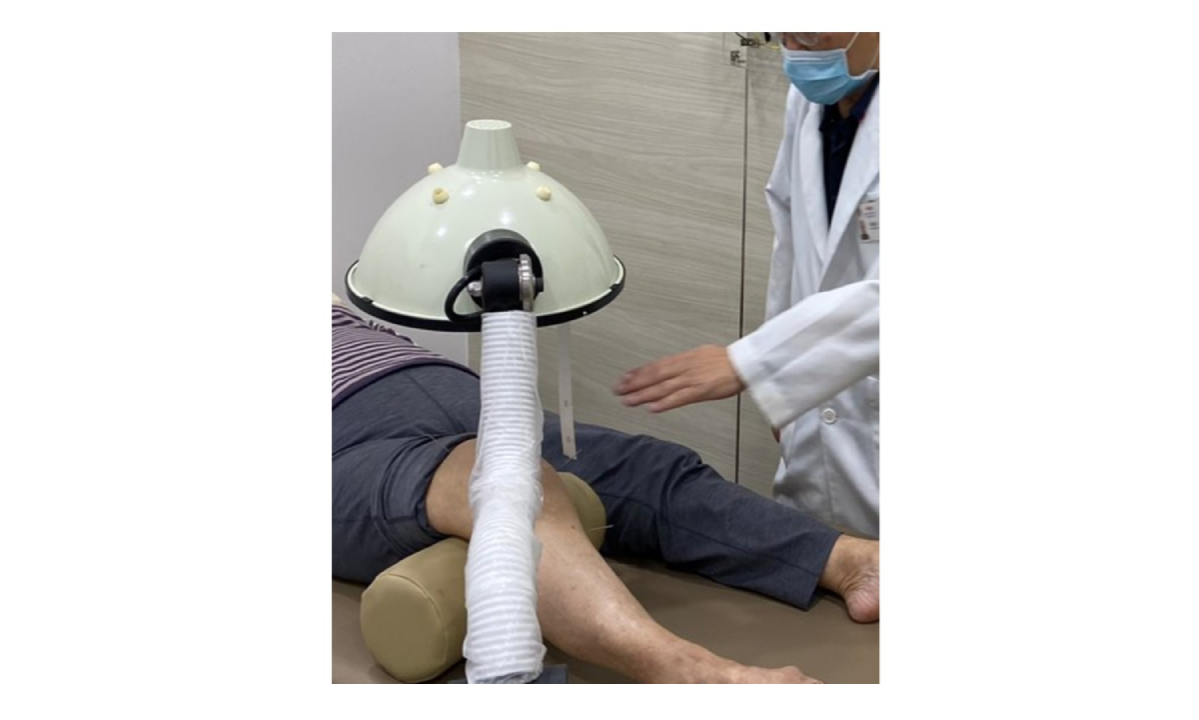
Heat therapy applied during acupuncture.

The patient will be treated in a supine position with pillows under both knees. After a standard disinfection procedure, a 30 × 40–mm disposable stainless steel acupuncture needle (YangTzeKing Impex S Pte Ltd) will be inserted according to the location defined; the direction of insertion and the safe depth range are specified in Multimedia Appendix 1, Figure S2 [21, 30,31]. Basic manipulations such as lifting and thrusting, twirling, and rotating are applied until the needling sensation (*de-qi*) is obtained. After *de-qi* is obtained, uniform reinforcing-reducing (平补平泻) manipulations are applied. The needles will be retained for 30 minutes (except for weizhong [BL40], where the needle is removed immediately after the needling sensation is obtained), whereupon 2 manipulations will be applied at 10-minute intervals before needle removal. During needle retention, far-infrared heat therapy will be applied at the lateral, medial, and anterior sides of the knee for 10 minutes each for a total of 30 minutes while maintaining a 30-cm safety distance (Figure 4). The treated region will be observed and checked for redness at 10-minute intervals to prevent burn injury. A total of 12 acupuncture sessions will be administered twice a week for 6 weeks in addition to standard care. A minimum of 50% attendance (6 of 12 sessions) is required to qualify as completing the study treatment. There are no make-up sessions should the patient miss the scheduled appointment. Total numbers and dates of attended and missed sessions will be recorded for analysis purposes. The study intervention will be conducted at TTSH, CIMC, or SCHMI. The patients are allowed to indicate their preference for the treatment location at the start of the intervention and must attend at the selected location throughout the 6-week intervention. Each TCM session takes approximately 40 minutes, involving acupuncture and heat therapy. Safety precautions will also be exercised to ensure the safety of the patients. Generally, acupuncture is safe and has a low risk of adverse events [37]. However, since the most common adverse events are subcutaneous hematomas and hemorrhages at the site of needling [37], the TCM practitioner will take extra precaution if patients indicate during informed consent that they are taking antiplatelets or anticoagulants. All adverse events that occur during the treatment will be documented.

#### Standard Care

Standard care generally constitutes simple lifestyle advice, analgesia, and a referral to the outpatient physiotherapist. The physiotherapist conducts an assessment and recommends a variety of lifestyle modifications and exercise therapy. Any other treatment or the involvement of allied health professionals, such as dieticians, will take place at the discretion of the managing physician. The patients in the standard care group will be discouraged from seeking acupuncture treatment for 6 to 8 weeks after randomization. However, if they do so, it will be noted and accounted for in the analysis.

#### Withdrawal Criteria

The study intervention may be discontinued under the following circumstances: (1) a serious adverse event arises from the intervention, (2) a participant opts for total knee replacement, (3) a participant is unable to continue the study (eg, because of the sudden onset of any medical condition that prohibits continuing the study), and (4) a collaborator withdraws due to unforeseen circumstances. The participants may also withdraw voluntarily from the study intervention for any reason.

#### Outcome Measures

The choice of outcome measures is based on the OARSI guidelines for lifestyle diet and exercise clinical trials in osteoarthritis [38]. The recommended core outcomes include pain, physical function, and global patient assessment. Additional outcomes include health-related QOL and global physician assessment. Outcome measures will be collected at baseline (pretreatment), week 6, week 12, and week 26. The primary end point in this study is the Knee Osteoarthritis Outcome Score (KOOS-12) at week 6. KOOS-12 is a specialized tool designed to evaluate patients’ knee condition, and it assesses the short- and long-term effects of knee injuries [39]. It reduces respondent burden by 70% from the original KOOS while providing scale scores for knee-specific pain, function, and QOL, along with a summary measure of overall knee impact [40]. The KOOS score has been validated in Singapore [39,41].

Baseline measures such as demographics, socioeconomic status, medical comorbidities, knee symptoms, radiographic severity, height, and weight will be collected.

Additionally, after considering the progression and severity of KOA as defined in TCM clinical guidelines published in China (Multimedia Appendix 1, Figure S3), 2 evaluation forms have been designed. The Knee Osteoarthritis TCM Evaluation Form (Multimedia Appendix 1, Figure S4) was specifically created to integrate the KOOS-12 knee survey with questions based on TCM syndrome differentiation for KOA. This will enable the investigators to assess the efficacy of the treatment by analyzing syndrome differentiation. This form will be administered at week 1 (start of treatment, before the first acupuncture session), week 4 (midtreatment, before the seventh acupuncture session) and week 6 (end of treatment, after the 12th or last acupuncture session). The Knee Osteoarthritis Visual Analog Scale and Range of Motion Evaluation Form (Multimedia Appendix 1, Figure S5) will be administered before and after each of the 12 biweekly acupuncture sessions over 6 weeks.

Clinical data (glycated hemoglobin, blood pressure, and other chronic disease parameters) and operational and cost data (health care use) will also be obtained through the health care administrative databases for economic evaluation.

#### Outcome Assessment and Blinding

Except for the TCM evaluation forms (Multimedia Appendix 1, Figures S4 and S5), the remaining outcome measures will be measured by blinded outcome assessors. All outcome assessors will receive training prior to study initiation to ensure good inter- and intraobserver reliability, particularly for the functional performance testing. Patients will be instructed not to reveal their allocation to the outcome assessors. Outcome assessment will be conducted at the TTSH physiotherapy clinic. Unblinding will be carefully considered and justified in each case of medical emergency or clinical treatment decisions that require the intervention to be known, following the study protocol and ethical guidelines.

#### Sample Size Calculation

The sample size needed to find a 10-point difference for KOOS-12 (the primary outcome) between the intervention and control arms with a power of 90%, a *P* value of .05 (2-sided) and an SD of 14 is n=42. The 10-point difference is based on the minimal clinical important difference for KOOS [42]. The anticipated baseline and SD are based on studies done in similar populations. Accounting for a 20% drop-out rate, a total of 100 patients will be needed for the study.

#### Data Analysis Plan

Results will be analyzed by the intention-to-treat principle. However, data will also be analyzed by a per-protocol approach to account for protocol violations, such as patients who were deemed not compliant to treatment or patients who underwent a surgical procedure of the knee due to treatment failure during the study. Descriptive frequency analysis will be used for baseline characteristics. For continuous variables, the mean and SD will be reported and for categorical variables, the frequencies and percentages will be reported. Between-group comparisons of change from baseline to the 26-week follow-up in the primary and secondary continuous outcomes will be analyzed using a generalized linear mixed model (GLMM). Testing for normality of distributions of outcomes will be based both on the Shapiro-Wilks test and a visual analysis of the histogram plot. Categorical secondary outcomes will be analyzed using the ordinal logistic regression function under the GLMM. A 2-sided *P* value less than .05 will be considered statistically significant. An analysis will be done on the nature of the missing data to determine if the data are missing at random or a systemic bias is present resulting from the missing data.

#### Data Management

All data will be monitored by the principal investigator and the study team independently of the study sponsor. Data quality measures include queries to identify outliers and missing data. A unique identifier will be assigned to each patient after enrollment to ensure patient confidentiality. Data will be collected and stored on the REDCap system, which is a widely used and secure web application for clinical data management in research. REDCap is password protected, and access rights to the study database are only granted to authorized people. Physical study files will be stored under lock and key.

#### Safety Monitoring Plan

A safety monitoring review will be done quarterly. Data monitored include serious adverse events and research-related events such as pain, burn, or any other adverse events that may arise due to the conduct of study activities. Safety monitoring reviews will be done by the principal investigator and the co–principal investigator of the study team. The study may be stopped by the principal investigator if serious safety concerns emerge. In the event that a critical adverse event occurs and a decision must be made to terminate the study, interim analyses will be conducted by an independent statistician.

Adverse events and other unanticipated effects of the intervention will be spontaneously reported by the acupuncturists. All reporting will be collected and assessed by the study team. Unanticipated problems involving risk to participants or other events will be reported to the institutional review board.

#### Confidentiality of Data and Patient Records

Only authorized study personnel will have access to the secured cabinet(s) or room(s) where hard-copy data will be stored. The electronic data will be stored on a password-protected desktop computer, laptop, and external HDD. The databases will not include subject identifiers, and the data relating subject identifiers to subject identification codes will be stored separately.The research data will only be accessible to the study team (including principal investigators, co-investigators, research coordinators, and statisticians). The anonymized and password-protected sharing of research data will be allowed with external institutions.

#### Ancillary and Posttrial Care

Enrolled participants are covered by indemnity for unexpected negligent harm due to the trial procedure.

#### Dissemination Plan

We anticipate that it will take between 3 and 4 months to compile the final results for the appropriate journal. The study results will be made available to the participating physicians and the medical and TCM practitioner community via academic publication.

### Component 2: Economic Evaluation

An economic evaluation will be conducted in tandem. The Panel on Cost-Effectiveness in Health and Medicine recommends the use of a societal perspective to ensure that potentially important indirect costs, such as productivity and caregiver cost, are not omitted [43]. The study will be conducted from a societal perspective to determine the cost-effectiveness of the intervention [43].

The cost data will be collected via hospital administrative databases and patient-reported questionnaires to estimate direct medical, direct nonmedical, and indirect costs based on the validated OA Cost and Consequences Questionnaire (OCC-Q), which has been adapted to the Singapore context. The OCC-Q will be administered at baseline, 12 weeks, and 26 weeks.

The primary measure of health benefit will be quality of life years measured using the EQ-5D [44]. The incremental cost-effectiveness ratio over the trial period of acupuncture with heat therapy in conjunction with standard care compared to standard care only will be determined.

Results from the economic evaluation will be reported based on the Consolidated Health Economic Evaluation Reporting Standards (CHEERS) statement [45].

### Component 3: Process Evaluation

The process evaluation will be embedded within the trial. The process evaluation is crucial in understanding the functioning of an intervention by examining its implementation, mechanisms of impact and contextual factors, and use of evaluation to understand how interventions work in practice. This is vital in building an evidence base that informs policy and practice. The Medical Research Council guidelines for the conduct of process evaluations [45] will be used to design the process evaluation.

The focus will be on fidelity of actual delivery, context, and mechanisms of impact with the goal of eventual large-scale implementation. Context includes anything external to the intervention that may act as a barrier or facilitator to its implementation. The examination of mechanisms of impact seeks to identify the potential causal pathways that resulted in the changes seen.

### Ethical Considerations

Ethics approvals were obtained from the National Healthcare Group Domain Specific Review Board (2021/01106) and Parkway Independent Ethics Committee (PIEC/2022/022), which oversee human research studies in the participating centers. This study will be conducted in accordance with the ethical principles in the Declaration of Helsinki, International Council for Harmonisation Good Clinical Practice guidelines, and it is consistent with the applicable regulatory requirements of the Human Biomedical Research Act in Singapore. Written informed consent will be obtained from all participants who agree to join the study after screening. A translator and an impartial witness will be present throughout the consent process if a participant is unable to understand English. All study documents were deidentified and research data will be anonymized and password-protected to ensure privacy and confidentiality. This trial is covered under the National Clinical Trials Insurance.

## Results

Patient enrollment has been ongoing since August 2022. The recruitment process is anticipated to conclude by July 2024, and the findings will be analyzed and publicized as they are obtained. As of November 6, 2023, our patient enrollment stands at 65 individuals.

## Discussion

### Overview

TCM has been growing in popularity as a viable treatment option for KOA in recent times. Systematic reviews have suggested that TCM has potential effectiveness in terms of pain relief and functional improvement and leads to few adverse events [46]. However, there have been significant concerns raised about the quality of evidence, with significant methodological flaws in many of the studies and a high risk of bias. Acupuncture in particular has had a fairly large base of evidence, but an overview of the various systematic reviews on the effectiveness and safety of acupuncture in KOA suggested while it has advantages in KOA, there is a significant risk of bias and reporting deficiencies that needs to be addressed [47].

In addition, there is little evidence for TCM treatment of KOA that is tailored to the unique demographics of populations in tropical climates. Many local practitioners often refer to China’s Clinical Guidelines of Acupuncture and Moxibustion for KOA, published in 2015 [20]. The guidelines include treatment options based on various TCM syndrome classifications, such as liver and kidney yin deficiency with blood stasis in tendons, spleen and kidney deficiency with dampness in the joint, and kidney yang deficiency with phlegm and blood stasis syndromes. Each syndrome delineates a distinct pattern of symptoms. A local study highlighted a significant difference in the syndrome differentiation for KOA (Multimedia Appendix 1, Figure S6 [48]), with a very high proportion (51%) of patients with KOA in Singapore experiencing the spleen and kidney deficiency with dampness in joint syndrome type, as compared to a mere 4.2% of patients with KOA in China [48,49]. The guidelines from China therefore may not be suited to Singapore’s unique patient demographics.

Our HARMOKnee study aims to (1) add high-quality evidence for acupuncture treatment for KOA to the existing literature on a broader scale; (2) support the development of locally standardized national evidence-based practice guidelines tailored to our unique population demographics; (3) support the implementation and integration of acupuncture and heat therapy into current treatment models supported by process and economic evaluations; and (4) establish a foundation for the development of an integrated model of care that harmonizes both Western and Eastern care components.

### Strengths and Limitations

One of the major strengths of our HARMOKnee study is that it follows the gold standard for research design: an RCT. The RCT design guarantees that the research uses a rigorous methodology and controls for potential confounding variables. The likelihood of bias can be reduced through allowing more precise assessment of the intervention’s efficacy. This study also adheres to the STRICTA guidelines, which provide a standardized framework for reporting acupuncture trials. All relevant data will be reported, thereby enhancing the study’s transparency and reproducibility. Moreover, this effectiveness-implementation hybrid study has incorporated an implementation component with the clinical effectiveness assessment. Its benefits include allowing the researchers to maximize the efficiency of their research while collecting data on both effectiveness and implementation, thereby identifying gaps in implementation and increasing the likelihood that the interventions will be effectively implemented and disseminated in real-world settings.

Limitations include the use of a pragmatic study design to mimic real-world conditions as closely as possible. This has an impact in terms of what treatment the control arm receives and how the intervention is delivered. Considerations to ensure intervention delivery fidelity have been incorporated, including standardization and training among acupuncturists. In addition, the treatment that the control arm receives will be meticulously recorded as part of the cost questionnaire and will be included in the analysis. Second, due to logistical and funding limitations, we are only able to include a follow-up period of up to 26 weeks. This will not allow us to establish the long-term effectiveness of this intervention.

### Conclusions

The findings of our HARMOKnee study will contribute substantial evidence to the current body of literature regarding the effectiveness of acupuncture treatment for KOA. Additionally, we aim to facilitate the creation of standardized national guidelines for evidence-based practice that are specifically tailored to our unique population demographics. Furthermore, we seek to promote the adoption and integration of acupuncture and heat therapy into existing treatment models.

## Appendix

Multimedia Appendix 1 HARMOKnee Study Protocol.

## Abbreviations

CHEERS: Consolidated Health Economic Evaluation Reporting Standards
CIMC: Complementary Integrative Medicine Clinic
CONSORT: Consolidated Standards of Reporting Trials
GLMM: generalized linear mixed model
HARMOKnee: Heat and Acupuncture to Manage Osteoarthritis of the Knee
KOA: knee osteoarthritis
KOOS-12: Knee Osteoarthritis Outcome Score
OARSI: Osteoarthritis Research Society International
OCC-Q: OA Cost and Consequences Questionnaire
PP: per-protocol
PRECIS-2: Pragmatic Explanatory Continuum Indicator Summary
QOL: quality of life
RCT: randomized controlled trial
REDCap: Research Electronic Data Capture
SCHMI: Singapore Chung Hwa Medical Institution
SPIRIT: Standard Protocol Items: Recommendations for Interventional Trials
STRICTA: Standards for Reporting Interventions in Clinical Trials of Acupuncture
TCM: traditional Chinese medicine

## Data Availability

The data that support the findings of this study are available on request from the corresponding author. The data are not publicly available due to restrictions on information that could compromise the privacy of research participants.
