# Supplementary material for: Heat and Acupuncture to Manage Osteoarthritis of the Knee (HARMOKnee): Protocol for an Effectiveness-Implementation Hybrid Randomized Controlled Trial
Source: JMIR Res Protoc. 2024 Apr 3;13:e54352. doi: 10.2196/54352 (PMC11024749; doi:10.2196/54352)
Supplement: Multimedia Appendix 1 [file resprot_v13i1e54352_app1.pdf]

Figure S1. Comparison of Protocols and Acupoints Across Different Acupuncture Randomised Controlled Trials

	Title	Subject	Control	Intervention	Frequency	Syndrome or meridian differentiation	ROM assessment	EA	Physio	Cupping
1	Effectiveness and feasibility of acupuncture for knee osteoarthritis: a pilot randomized controlled trial[21]	knee osteoarthritis (Kellgren grade II or III)	Sham acupoints (7 pts, not specific) [n=21]	Local + distal acupoints (8-10 pts) [n=21]	3 per wk x 8 wks = 24 20min per session	NO	NO	NO	NO	NO
		Conclusion: Patients with knee osteoarthritis who received traditional Chinese acupuncture had not significantly less pain and better function than did patients who received sham acupuncture either after treatments or 26 weeks later								
2	Effect of acupuncture therapies combined with usual medical care on knee osteoarthritis[10]	knee osteoarthritis (Kellgren grade II or III)	Usual care (UC): Western oral med [n=30]	1.Manual Acu +UC [n=30] 2.EA +UC [n=30] dilatational wave, 2/100Hz, 0.2 ms pulse width	5 per wk x 10 wks =50 30min per session	NO	NO	YES	NO	NO
		Conclusion: AP + UC or EA + UC group demonstrating remarkable advantages compared with the UC group. However, the difference between the two acupuncture therapies combined with usual pharmacotherapies was not obvious in the treatment of KOA patients								
3	Efficacy of electro-acupuncture and manual acupuncture versus sham acupuncture for KOA: study protocol for a randomised controlled trial[22]	knee osteoarthritis (Kellgren grade II or III)	Sham acupuncture (8 pts) [n=160]	1.Manual acu [n=160] 2.EA [n=160] Primary acupoints + Secondary acupoints (based on meridians)	3 per wk x 8 wks = 24 30min per session	Meridian	NO	YES	NO	NO
		Conclusion: Not available								
4	Early intervention of Knee Osteoarthritis using acupuncture: a randomised controlled trial[23]	Unilateral knee osteoarthritis at early stage (Kellgren grade 0 or I), <1mth	Usual care (UC): Western Topical Med + patient education [n=30]	1.Manual Acu +UC [n=30]	3 per wk x 2 wks = 6 30min per session	NO	NO	NO	NO	NO
		Conclusion: No significance between the 2 group								

Title	Subject	Control	Intervention	Frequency	Syndrome or meridian differentiation	ROM assessment	EA	Physio	Cupping	Title
5	Acupuncture as an adjunct to exercise-based physiotherapy for osteoarthritis of the knee: randomised controlled trial[24]	knee osteoarthritis	Physio [n=116]	1.Manual Acu + Physio [n=117]<br>2.Sham Acu + Physio pn=119]<br>6 local +10 distal pts	10 times<br>25-35min per session<br><br>Physio: 6 sessions x 30 min per session	NO	NO	NO	YES	NO
		The addition of acupuncture to a course of advice and exercise for osteoarthritis of the knee delivered by physiotherapists provided no additional improvement in pain scores.								
6	Acupuncture combined with cinesiotherapy cupping for knee osteoarthritis with qi stagnation and blood stasis syndrome: a randomized controlled trial[25]	knee osteoarthritis with qi stagnation & blood stasis	Acu + Cupping [n=39]	Acu + Cupping + exercise [n=39]<br><br>9 pts	10 sessions x 3 cycles with 2 days interval in between (34 days)	Syndrome	NO	NO	YES	YES
		Intervention group has better efficacy than control group								
7	Severe knee osteoarthritis: a randomized controlled trial of acupuncture, physiotherapy (supervised exercise) and standard management for patients awaiting knee replacement[26]	Patients awaiting knee arthroplasty	home exercise + advice leaflet [n=61]	1.Acu [n=60]<br>2.Physio [n=60]	Once/wk x 6 wks<br>20min per session (acu)<br>1 hr per session (physio)	NO	NO	NO	YES	NO
		Patients with severe knee osteoarthritis can achieve a short-term reduction in OKS when treated with acupuncture. However, we failed to demonstrate any other clinically or statically significant effects between the groups.								
8	Acupuncture treatment for knee osteoarthritis with sensitive points: protocol for a multicentre randomised controlled trial[27]	mild or moderate KOA	No acupuncture (Std acupuncture on study completion) [n=666]	1.acu at high-sensitive points [n=666]<br>2.acu at low-sensitive points [n=666]	3 per wk x 4 wks = 12<br>30min per session	NO	NO	NO	NO	NO
		Conclusion: Not available								

Title	Subject	Control	Intervention	Frequency	Syndrome or meridian differentiation	ROM assessment	EA	Physio	Cupping	Title
9	Acupuncture of different treatment frequency in knee osteoarthritis: a protocol for a pilot randomized clinical trial[28]	knee osteoarthritis (Kellgren grade II or III)	No control	1.Acu at 3 times per week [n=30]<br>2.Acu at 1 time per week [n=30]<br>X 8 sessions<br>Give paracetamol when pain > 8<br>dilatational wave, 2/100Hz, 0.1-1 ms pulse width	According to intervention	Meridian	NO	YES	NO	NO
		Conclusion: Not available								
10	Integrating Acupuncture With Exercise-Based Physical (EPT) Therapy for Knee Osteoarthritis: A Randomized Controlled Trial[29]	knee osteoarthritis (Kellgren grade II or III), > 6 mth	EPT + Sham acu	EPT + Manual acu	1-2 x per week x 12 sessions 20min per session	NO	NO	NO	YES	NO
		Puncturing acupuncture needles did not perform any better than non-puncturing needles integrated with EPT.								

References:

[10]Zhang L, Yuan H, Zhang L, Li J, Li H. Effect of acupuncture therapies combined with usual medical care on knee osteoarthritis. J Tradit Chin Med. 2019 2019/02//;39(1):103-10

[21] Lin LL, Li YT, Tu JF, Yang JW, Sun N, Zhang S, et al. Effectiveness and feasibility of acupuncture for knee osteoarthritis: a pilot randomized controlled trial. Clin Rehabil. Dec 2018;32(12):1666-1675.

[22]Tu JF, Yang JW, Lin LL, Wang TQ, Du YZ, Liu ZS, et al. Efficacy of electro-acupuncture and manual acupuncture versus sham acupuncture for knee osteoarthritis: study protocol for a randomised controlled trial. Trials. Jan 25, 2019;20(1):79.

[23]Luo X, Hou XS, Tian ZY, Meng X, Li SM, Bai P. [Randomized controlled clinical trial of acupuncture treatment for knee osteoarthritis in the early stage]. Zhen Ci Yan Jiu. Mar 25, 2019;44(3):211-215

[24]Foster NE, Thomas E, Barlas P, Hill JC, Young J, Mason E, et al. Acupuncture as an adjunct to exercise based physiotherapy for osteoarthritis of the knee: randomised controlled trial. BMJ. Sep 01, 2007;335(7617):436.

[25]Qiu JQ, Liu SR, Lin QL, Li MJ, Zhuang JX, Wu GW. [Acupuncture combined with cinesiotherapy cupping for knee osteoarthritis with stagnation and blood stasis syndrome: a randomized controlled trial]. Zhongguo Zhen Jiu. May 12, 2019;39(5):462-466.

[26]Williamson L, Wyatt MR, Yein K, Melton JTK. Severe knee osteoarthritis: a randomized controlled trial of acupuncture, physiotherapy (supervised exercise) and standard management for patients awaiting knee replacement. Rheumatology (Oxford). Sep 2007;46(9):1445-1449.

[27]Tang L, Jia P, Zhao L, Kang D, Luo Y, Liu J, et al. Acupuncture treatment for knee osteoarthritis with sensitive points: protocol for a multicentre randomised controlled trial. BMJ Open. Oct 02, 2018;8(10):e023838.

[28]Lin LL, Tu JF, Shao JK, Zou X, Wang TQ, Wang LQ, et al. Acupuncture of different treatment frequency in knee osteoarthritis: a protocol for a pilot randomized clinical trial. Trials. Jul 11, 2019;20(1):423.

[29]Chen LX, Mao JJ, Fernandes S, Galantino ML, Guo W, Lariccia P, et al. Integrating acupuncture with exercise-based physical therapy for knee osteoarthritis: a randomized controlled trial. J Clin Rheumatol. Sep 2013;19(6):308-316.

The acupoints used in the protocols of these several RCTs were considered in our intervention

= Primary Acupoints ; = Secondary Acupoints

	Title	Liangqiu ST34	Dubi ST35	Zusanli ST36	Heding Ex-LE2	Neixiyan EX-LE5	Xiyangguan GB33	Yanglingquan GB34	Yinlingquan SP9	Xuehai SP10	Ququan LR8	Fengshi GB31	Liangqiu ST34	Xuanzhong GB39	Zulingqi GB41	Fenglong ST 40	Jiexi ST41	Taichong LR3	Kunlun BL60	Sanyinjiao SP6	Taixi KI3	Hegu LI4	Ququan LR8	Futu ST32	Weizhong BL40	Ashi points
1	Effectiveness and feasibility of acupuncture for knee osteoarthritis: a pilot randomized controlled trial	Any 5-6 pts											Any 8-10 pts													
2	Effect of acupuncture therapies combined with usual medical care on knee osteoarthritis																									
3	Efficacy of electro-acupuncture and manual acupuncture versus sham acupuncture for KOA: study protocol for a randomised controlled trial																									
		anterior aspect of the affected knee joint (yangming meridian syndrome). Three adjunct acupoints will be chosen from futu (ST32), liangqiu (ST34), heding (EX-LE2), zusanli (ST36) and fenglong (ST40).<br>medial aspect of the affected knee joint (three-yin meridian syndrome) Three adjunct acupoints will be chosen from xuehai (SP10), yingu (KI10), yinlingquan (SP9), xiguan (LR7), sanyinjiao (SP6), taixi (KI3), taichong (LR3) and gongsun (SP4).<br>posterior aspect of the affected knee joint (taiyang meridian syndrome). Three adjunct acupoints will be chosen from weiyang (BL39), weizhong (BL40), chengshan (BL57) and kunlun (BL60).<br>lateral aspect of the affected knee joint (shaoyang meridian syndrome). Three adjunct acupoints will be chosen from fengshi (GB31), yanglingquan (GB34), waiqiu (GB36), xuanzhong (GB39) and zulinqi (GB41).<br>If more than two aspects are affected, three adjunct acupoints will be chosen from those for the relevant syndromes																								
4	Early intervention of Knee Osteoarthritis using acupuncture: a randomised controlled trial																									
5	Acupuncture as an adjunct to exercise-based physiotherapy for osteoarthritis of the knee: randomised controlled trial																									
		Six and 10 acupuncture points from 16 commonly used local and distal points were selected. Local points were Sp 9, Sp 10, St 34, St 35, St 36, Xiyan, Gb 34, and trigger points. Distal points were LI 4, TH5, Sp 6, Liv 3, St 44, Ki 3, Bl 60, and Gb 41.																								

	Title	Liangqiu ST34	Dubi ST35	Zusanli ST36	Heding Ex-LE2	Neixiyan EX-LE5	Xiyangguan GB33	Yanglingquan GB34	Yinlingquan SP9	Xuehai SP10	Ququan LR8	Fengshi GB31	Liangqiu ST34	Xuanzhong GB39	Zulingqi GB41	Fenglong ST 40	Jiexi ST41	Taichong LR3	Kunlun BL60	Sanyinjiao SP6	Taixi KI3	Hegu LI4	Ququan LR8	Futu ST32	Weizhong BL40	Ashi points
6	Acupuncture combined with cinesiotherapy cupping for knee osteoarthritis with qi stagnation and blood stasis syndrome: a randomized controlled trial		■	■	■	■		■	■	■			■	■												
7	Severe knee osteoarthritis: a randomized controlled trial of acupuncture, physiotherapy (supervised exercise) and standard management for patients awaiting knee replacement		■	■		■		■	■	■								■								
8	Acupuncture treatment for KOA with sensitive points…		■	■	■	■		■	■	■			■										■		■	
		Also include Yingu (KI10), Xiguan (LR7), Weiyang (BL39).																								
9	Acupuncture of different treatment frequency in KOA: …		■			■	■															■				■
		The selection of the three customized acupoints will be based on the experience of the acupuncturist. Therefore, the number of needles used is eight (unilateral). Ie ST34, ST36, ST32, ST40 and EX-LE2 will be used for pain at the stomach meridian. GB31, GB36, GB34, GB39 and GB41 will be used for pain at the gallbladder meridian. BL39, BL40, BL57 and BL60 will be used for pain at the bladder meridian. LR7, SP9, SP10, KI10, SP4, SP6, LR3 and KI3 will be used for pain at the three-yin meridians of the foot.																								
10	Integrating Acupuncture With Exercise-Based Physical (EPT) Therapy for KOA:…		■	■		■		■	■					■					■	■	■					

Figure S2. Meridian Differentiation in KOA and acupoints selected

According to the Meridian Theory, all six meridians of the foot travel through the knees. Figures adapted from 'A Manual of Acupuncture' by Deadman P, Baker K, Al-Khafaji M, 2007.

Table 1. Meridians of the foot

Lateral position	Medial position	Posterior position	Anterior position
The Gallbladder meridian of Foot-Shaoyang	The Spleen meridian of Foot-Taiyin,<br>The Liver meridian of Foot-Jueyin,<br>The Kidney meridian of Foot-shaoyin	The Bladder meridian of Foot-Taiyang	The Stomach meridian of Foot-Yangming
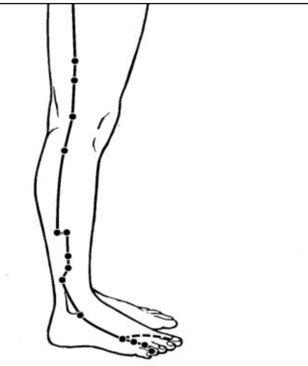	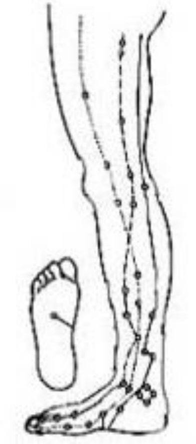	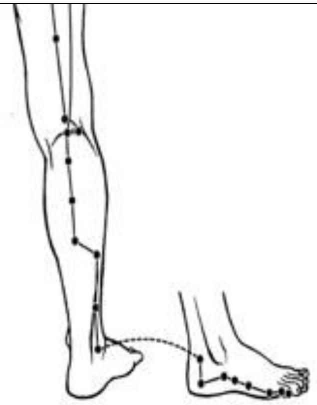	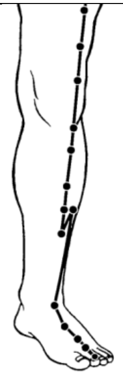

Table 2. Selected Acupoints for the Intervention Group

Acupoints	Position around the knee/elbow	Meridians	Location[30,31]	Safety depth [direction][21]
**Proximal acupoints (near the knee)**				
1. Yinlingquan SP9	Medial	Spleen meridian of Foot-Taiyin [He-Sea point]	On the tibial aspect of the leg, in the depression between the inferior border of the medial condyle of the tibia and the medial border of the tibia.	1-1.5 cun [perpendicular]
2. Zusanli ST36	Anterior	Stomach meridian of Foot-yangming [He-Sea point]	On the anterior aspect of the leg, on the line connecting ST35 with ST41, 3 B-cun inferior to ST35. Note: ST36 is located on the tibialis anterior muscle.	1-1.5 cun [perpendicular]
3. Ququan LR8	Medial	Liver Meridian of Foot Jueyin [He-Sea point]	On the medial aspect of the knee, in the depression medial to the tendons of the semitendinosus and the semimembranosus muscles, at the medial end of the popliteal crease.	1-1.5 cun [perpendicular]
4. Yanglingquan GB34	Lateral	Gallbladder Meridian of Foot Shaoyang [He-Sea point]	On the fibular aspect of the leg, in the depression anterior and distal to the head of the fibula.	1-1.5 cun [perpendicular]
5. Yingu KI10	Posteromedial	Kidney Meridian of Foot-Shaoyin [He-Sea point]	On the posteromedial aspect of the knee, just lateral to the semitendinosus tendon, in the popliteal crease.	1-1.5 cun [perpendicular]
6. Weizhong BL40	Posterior	Bladder Meridian of Foot-Taiyang [He-Sea point]	On the posterior aspect of the knee, at the midpoint of the popliteal crease.	1-1.5 cun [perpendicular]
7. Liangqiu ST34	Anterior	Stomach meridian	On the anterolateral aspect of the thigh, between the vastus lateralis muscle and the lateral border of the rectus femoris tendon, 2 B-cun superior to the base of the patella.	1-1.5 cun [perpendicular]
8. Xuehai SP10	Medial	Spleen meridian	On the anteromedial aspect of the thigh, on the bulge of the vastus medialis muscle, 2 B-cun superior to the medial end of the base of the patella.	1-1.5 cun [perpendicular]
**Distal acupoints (near the elbow)**				
1. Chize LU5	Anterior	Lung Meridian [He-Sea point]	On the anterior aspect of the elbow, at the cubital crease, in the depression lateral to the biceps brachii tendon.	0.8-1.2 cun [perpendicular]
2. Quchi LI11	Lateral	Large Intestine Meridian [He-Sea point]	On the lateral aspect of the elbow, at the midpoint of the line connecting LU5 with the lateral epicondyle of the humerus.	1-1.5 cun [perpendicular]
3. Shaohai HT3	Antero-medial	Heart Meridian [He-Sea point]	On the anteromedial aspect of the elbow, just anterior to the medial epicondyle of the humerus, at the same level as the cubital crease.	0.5-1 cun [perpendicular]
4. Xiaohai SI8	Posteromedial	Small Intestine Meridian [He-Sea point]	On the posteromedial aspect of the elbow, in the depression between the olecranon and the medial epicondyle of the humerus bone.	0.3-0.5 cun [perpendicular]

5. Quze PC3	Anterior	Pericardium Meridian [He-Sea point]	On the anterior aspect of the elbow, at the cubital crease, in the depression medial to the biceps brachii tendon.	1-1.5 cun [perpendicular]
6. Tianjin TE10	Posterior	Triple Energizer (Sanjiao) Meridian [He-Sea point]	On the posterior aspect of the elbow, in the depression 1 B-cun proximal to the prominence of the olecranon.	0.5-1 cun [perpendicular]

Figure S3. Progression and Severity of Knee OA defined in the TCM Clinical Guidelines published in China[21]

These guidelines were subsequently used to help in the development of the TCM evaluation forms (Figure S4 an S5)

.

Table 1: Progression of KOA and Symptoms Observed at Each Stage, as Defined in TCM Clinical Guidelines

Symptoms	Early stage of KOA	Mid stage of KOA	Advanced stage of KOA
Knee pain	Medial pain is more common, especially climbing stairs or standing up	Moderate pain	Severe pain, requires walking tools or unable to walk.
Joint deformity	Not significant	Varus knee, knee flexion deformity	Significant Varus knee, knee flexion deformity
Local swelling	Not significant	Significant	
Tenderness at palpation	At the knee joint space and surrounding area when pressure is applied	Pain when pressure is applied	Pain when pressure is applied
Patellar grind test (Clarke's test)	Positive	Positive	Positive
Joint movement	Not affected	Limited joint movement. Unstable	Limited joint movement. Severely unstable
Radiologic evaluation	0 ~ I	II ~ III	IV

Table 2: Symptoms of Varying Severity of KOA (Mild, Moderate and Severe), as Defined in TCM Clinical Guidelines

Symptoms	Mild	Moderate	Severe
Pain/discomfort during sleep	Some discomfort, disappear after some activities	Pain may occur sometimes	Persistent pain
Pain increased in the morning exercise or when awake	Some discomfort, relieved after some activities	Some discomfort, relieved after some activities	Significant pain, do not relieve after some activities
Pain/discomfort when walking	Occur after long distance > 1km	Occur after short distance < 1km	Increased pain when walking
Pain/discomfort when standing up from sitting position	Mild	Insignificant, no assistance needed	Significant, assistance needed
Maximum walking distance (may be with pain)	> 1km but limited	0.3-1 km	<0.3 km
Daily activities	Perform with difficulty Occasionally	Perform with difficulty	Unable to perform
Climbing up or down the stairs	Able to perform	Perform with difficulty	Unable to perform
Squatting or bending knee	Able to perform	Perform with difficulty	Unable to perform
Walking on uneven road	Able to perform	Perform with difficulty	Unable to perform

Figure S4. Knee Osteoarthritis TCM Evaluation Form

The Knee Osteoarthritis TCM Evaluation Form is designed on the basis of KOOS-12 Knee Survey. In addition to the questions from KOOS-12 (highlighted in grey), questions according to TCM syndrome differentiation of KOA in Singapore are included. The final score will be combined to evaluate the outcome.

**Study Title**			
**Study ID**		**Date (DD/MM/YYYY)**	
**Gender**	☐ Male<br>☐ Female	**Attending Physician**	
**Age**		**Final Score (P+F+Q)**	

Kindly evaluate the symptoms and indicate the score for each item

Pain

No.	Symptoms	0 (No symptom)	1 (Mild)	2 (Moderate)	3 (Severe)	4 (extreme)	Score
P1	Pain scale (VAS)	0	1-3	4-5	6-8	9-10	
P2	Fixed, sharp pain	None	Mild	Moderate	Severe	Extreme	
P3	Dull pain	None	Mild	Moderate	Severe	Extreme	
P4	Pulling pain	None	Mild	Moderate	Severe	Extreme	
P5	Aching pain	None	Mild	Moderate	Severe	Extreme	
P6	Pain/discomfort during sleep	None	Mild	Moderate	Severe	Extreme	
P7	Increasing pain during rainy days	None	Mild	Moderate	Severe	Extreme	
P8	Increasing pain due to coldness	None	Mild	Moderate	Severe	Extreme	
P9	How often do you experience knee pain	None	Monthly	Weekly	Daily	Always	
What amount of knee pain have you experienced the last week during the following activities?							
P10	Walking on a flat surface	None	Mild	Moderate	Severe	Extreme	
P11.1	Going up stairs	None	Mild	Moderate	Severe	Extreme	
P11.2	Going down stairs	None	Mild	Moderate	Severe	Extreme	
P12.1	Sitting	None	Mild	Moderate	Severe	Extreme	
P12.2	Lying	None	Mild	Moderate	Severe	Extreme	
						Pain Score (P)	

Function, daily living

No.	Symptoms	0 (No symptom)	1 (Mild)	2 (Moderate)	3 (Severe)	4 (extreme)	Score
The following questions concern your physical function. By this we mean your ability to move around and to look after yourself. For each of the following activities please indicate the degree of difficulty you have experienced in the last week due to your knee.							
F1	Rising from sitting	None	Mild	Moderate	Severe	Extreme	
F2	Standing	None	Mild	Moderate	Severe	Extreme	
F3.1	Getting in a car	None	Mild	Moderate	Severe	Extreme	
F3.2	Getting out of a car	None	Mild	Moderate	Severe	Extreme	
F4	Twisting/pivoting on your injured knee	None	Mild	Moderate	Severe	Extreme	
F5	Stiffness after long sitting, lying or rest	Never	Rarely	Occasionally	Most of the time	All the time	
F6	Morning stiffness	Never	Rarely	Occasionally	Most of the time	All the time	
F7	Squatting or bending knee	Able to perform	Perform with slight difficulty	Unable to bend > 90°	Unable to bend > 45°	Unable to perform	
F8	Knee limited range of motion	None	Knee flexion <120°	Knee flexion <110°	Knee flexion <100°	Knee flexion <90°	
						Functional Score (F)	

Quality of life

No.	Symptoms	0 (No symptom)	1 (Mild)	2 (Moderate)	3 (Severe)	4 (extreme)	Score
Q1	How often are you aware of your knee problem?	None	Monthly	Weekly	Daily	Always	
Q2	Have you modified your life style to avoid potentially damaging activities to your knee?	None	Mild	Moderate	Severe	Extreme	
Q3	How much are you troubled with lack of confidence in your knee?	None	Mild	Moderate	Severe	Extreme	
Q4	In general, how much difficulty do you have with your knee?	None	Mild	Moderate	Severe	Extreme	
Q5	Weak and sore back	None	Rarely	Occasionally	Most of the time	All the time	
Q6	Body numbness, weak limbs	None	Rarely	Occasionally	Most of the time	All the time	
Q7	Dizziness	None	Rarely	Occasionally	Most of the time	All the time	
Q8	Aversion to cold and preferred warm	None	Rarely	Occasionally	Most of the time	All the time	
Q9	Warm sensation at chest, soles and palms	None	Rarely	Occasionally	Most of the time	All the time	
Q10	Night Sweat	None	Rarely	Occasionally	Most of the time	All the time	
Q11	Stomach distension	None	Rarely	Occasionally	Most of the time	All the time	
Q12	Loose stool	None	Rarely	Occasionally	Most of the time	All the time	
						Quality Score (Q)	

Tongue observation

1	Tongue color	☐Pale white	☐Pale red	☐Red	☐Dark red	☐Dark purplish
2.1	Tongue coating color	☐None	☐White	☐Yellowish-White	☐Yellow	☐Grey
2.2	Tongue coating thickness	☐None	☐Thin	☐Thick	☐Sticky	☐Thick & sticky

**Syndrome differentiation (To be evaluated by TCMP/Acupuncturist)**				
**Syndrome**	**Primary symptoms**	**Secondary symptoms**	**Tongue & pulse**	
Liver & Kidney yin deficiency with blood stasis in tendons	Dull pain or fixed sharp pain. Unfavourable knee flexion & extension, knee joint deformity, weak & soreness in lower back	Crepitus, pain increase during activity, muscle atrophy. Dizziness, warm palms and soles, night sweat.	Pale or purplish & dull tongue with thin, white coating.<br>Fine or wiry, fine pulse	
Spleen & Kidney deficiency with dampness in the joint	Dull pain, knee swelling, morning stiffness, body numbness, weak limbs	Pain aggravated during rainy days, cold limbs, abdominal distention, loose stools, frequent urination with increased volume	Pale, fat tongue with teeth marks, white, slippery or white, thick coating.<br>Fine or slippery pulse	
Kidney yang deficiency with phlegm & blood stasis	Dull pain or pulling pain at fixed location. Pain aggravated during night. Lassitude	Knee swelling, darken or dark red colour around the knee, unfavourable knee flexion & extension. Aversion to cold and preferred warm. Frequent urination	Pale, fat tongue with bruising spots or pale & dull tongue. Thin, white coating.<br>Sunken, fine pulse or fine, sluggish pulse	

Figure S5. Knee Osteoarthritis Visual Analog Scale & Range of Motion Evaluation Form

**Study Title**			
**Study ID**		**Date (DD/MM/YYYY)**	
**Gender**	Male Female	**Attending Physician**	
**Age**		**No. of days before pain relapse after last treatment**	

1. Visual Analog Scale (VAS)

**Pain before acupuncture (VAS1)**	☐ 0 No pain<br>☐ 2 Mild<br>☐ 4 Moderate	☐ 6 Severe<br>☐ 8 Very severe<br>☐ 10 Worst pain possible
**Pain after acupuncture (VAS2)**	☐ 0 No pain<br>☐ 2 Mild<br>☐ 4 Moderate	☐ 6 Severe<br>☐ 8 Very severe<br>☐ 10 Worst pain possible
**VAS2-VAS1**		

2. Active & passive range of motion

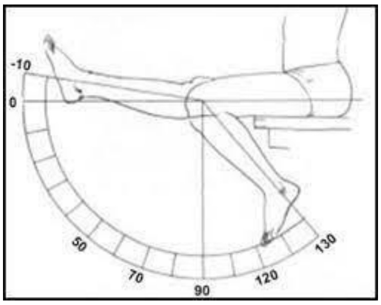

Knee flexion before acupuncture		Knee flexion after acupuncture	
Active ROM (Flex-AROM1)		Active ROM (Flex-AROM2)	
Passive ROM (Flex-PROM1)		Passive ROM (Flex-PROM2)	
Knee extension before acupuncture		Knee extension after acupuncture	
Active ROM (Ext-AROM1)		Active ROM (Ext-AROM2)	
Passive ROM (Ext-PROM1)		Passive ROM (Ext-PROM2)	

Flex-AROM2 - Flex-AROM1	
Flex-PROM2 - Flex-PROM1	
Ext-AROM2 - Ext-AROM1	
Ext-PROM2 - Ext-PROM1	

Figure S6. Symptoms, Tongue Appearance and Pulse Readings according to Syndrome differentiation of Knee Osteoarthritis (KOA) in Singapore[48]

Table 1. Syndrome differentiation of Knee Osteoarthritis (KOA) in Singapore

Syndrome	Primary symptoms	Secondary symptoms	Tongue & Pulse
Liver & Kidney yin deficiency with blood stasis in tendons	Dull pain or fixed sharp pain. Unfavourable knee flexion & extension, knee joint deformity, weak & soreness in lower back	Crepitus, pain increase during activity, muscle atrophy. Dizziness, warm palms and soles, night sweat.	Pale or purplish & dull tongue with thin, white coating. Fine or wiry, fine pulse
Spleen & Kidney deficiency with dampness in the joint	Dull pain, knee swelling, morning stiffness, body numbness, weak limbs	Pain aggravated during rainy days, cold limbs, abdominal distention, loose stools, frequent urination with increased volume	Pale, fat tongue with teeth marks, white, slippery or white, thick coating. Fine or slippery pulse
Kidney yang deficiency with phlegm & blood stasis	Dull pain or pulling pain at fixed location. Pain aggravated during night. Lassitude	Knee swelling, darken or dark red colour around the knee, Unfavourable knee flexion & extension. Aversion to cold and preferred warm. Frequent urination	Pale, fat tongue with bruising spots or pale & dull tongue. Thin, white coating. Sunken, fine pulse or fine, sluggish pulse

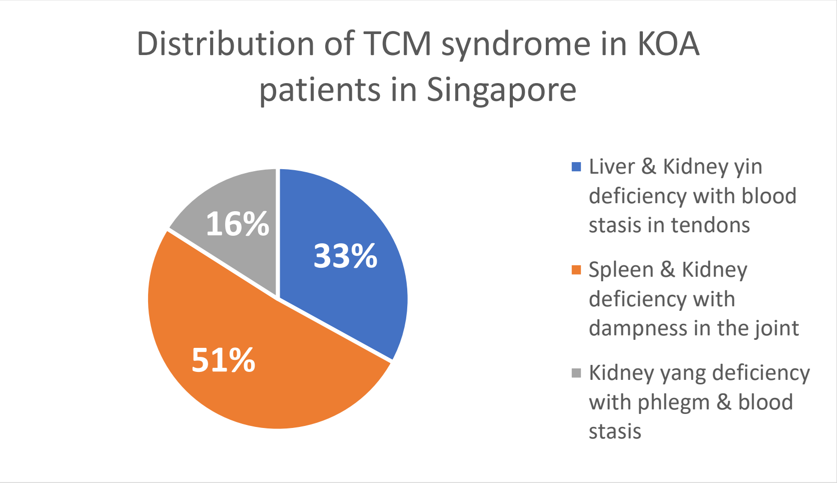

[48] Cai H. xin jia po xi guan gu xing guan jie yan (KOA) zhong yi zheng xing fen bu yan jiu [TCM syndrome distribution study of Knee Osteoarthritis in Singapore]. Tong Ji Yi Yao Journal. 2018;6:p9-12
